# Supplementary material for: Exploring the status of brucellosis in pregnant women presented with febrile illness at different healthcare facilities of Vehari and Lodhran zones of Pakistan
Source: PLoS Negl Trop Dis. 2025 Sep 15;19(9):e0013525. doi: 10.1371/journal.pntd.0013525 (PMC12448985; doi:10.1371/journal.pntd.0013525)
Supplement: Attachment — Submitted filename: Response to Reviewers Comments - Copy.docx [file pntd.0013525.s003.docx]

**Ref: Manuscript ID: PNTD-D-25-01396**

**Title:	Exploring the status of brucellosis in pregnant women presented with febrile illness at different healthcare facilities of Vehari and Lodhran zones of Pakistan**

**Responses to Reviewers’ Comments**

**Dear Editor-in-Chief**

PLOS Neglected Tropical Diseases

We really appreciate the worthy Editors / reviewers for pointing out some important shortcomings/deficiencies in the manuscript that helped us to improve its quality. Accordingly, manuscript has been revised as follows: The correction have been highlighted as yellow.

**Details are as follows:**

**Responses to Reviewer’s Comments (as mentioned in attached file)**

| **Sr. No.** | **Reviewer’s Comments** | **Response** |
| --- | --- | --- |
| 1. | The worthy reviewer has suggested changes in original manuscript as yellow highlighted areas. | All suggested changes have been incorporated as suggested by the worthy reviewer to improve the quality of manuscript. |

**Responses to Editor’s Comments**

| **Sr. No.** | **Editor’s Comments** | **Response** |
| --- | --- | --- |
| 1. | Lines 67 to 70: Please divide the long sentence into 2-3 separate sentences. | Modified as suggested by the worthy reviewer. |
| 2. | Line 73: Reference is needed. | References (No. 1 and 2) have been added in the manuscript as follows:  Murhekar MM, Singh R, Dwivedi GR, Gulafshan S. Perspective Chapter: Zoonosis – Brucellosis a Clinically Relevant Bacterial Disease. In: Rodriguez-Morales AJ, ed. *Current Topics in Zoonoses*. IntechOpen; 2024.  Rossetti CA, Arenas-Gamboa AM, Maurizio E. Caprine brucellosis: A historically neglected disease with significant impact on public health. *PLoS Negl Trop Dis*. Aug 2017;11(8):e0005692. doi:10.1371/journal.pntd.0005692 |
| 3. | Line 77: Quantify loss with a reference. | The suggested information has been added in the manuscript with relevant citations (No. 5 and 6).  Singh BB, Dhand NK, Gill JPS. Economic losses occurring due to brucellosis in Indian livestock populations. *Preventive Veterinary Medicine*. 2015/05/01/ 2015;119(3):211-215. doi:<https://doi.org/10.1016/j.prevetmed.2015.03.013>  MN, Boyle SM, Sriranganathan N. Brucellosis: A re-emerging zoonosis. *Veterinary Microbiology*. 2010/01/27/ 2010;140(3):392-398. doi:<https://doi.org/10.1016/j.vetmic.2009.06.021> |
| 4. | Line 79: Need recent reference. | As suggested by the reviewer, a recent reference (No. 7) has been added in the manuscript as follows:  Jamil T, Khan AU, Saqib M, et al. Animal and Human Brucellosis in Pakistan. Review. *Frontiers in Public Health*. 2021-July-30 2021;Volume 9 - 2021doi:10.3389/fpubh.2021.660508. |
| 5. | Line 93: The reference is not appropriate. | The most relevant reference (No. 7) containing mentioned information has been added in revised manuscript as follows:  Jamil T, Khan AU, Saqib M, et al. Animal and Human Brucellosis in Pakistan. Review. Frontiers in Public Health. 2021-July-30 2021;Volume 9 - 2021doi: https://doi.org/10.3389/fpubh.2021.660508 |
| 6. | Line 97: The reference is not accessible. | The updated references (11 and 12) have been added which are accessible online and are as follows:  Franc KA, Krecek RC, Häsler BN, Arenas-Gamboa AM. Brucellosis remains a neglected disease in the developing world: a call for interdisciplinary action. *BMC Public Health*. 2018/01/11 2018;18(1):125. doi: <https://doi.org/10.1186/s12889-017-5016-y>  Zhang N, Huang D, Wu W, et al. Animal brucellosis control or eradication programs worldwide: A systematic review of experiences and lessons learned. *Preventive Veterinary Medicine*. 2018/11/15/ 2018;160:105-115. doi:<https://doi.org/10.1016/j.prevetmed.2018.10.002> |
| 7. | Line 103: Several references have been cited, but not all references report the same data. | The irrelevant references citing the said information have been removed. The original article reporting the same data has been added as follows:  “Zheng R, Xie S, Lu X, Sun L, Zhou Y, Zhang Y, et al. A systematic review and meta-analysis of epidemiology and clinical manifestations of human brucellosis in China. Biomed Res Int. 2018;2018:5712920. doi: 10.1155/2018/5712920” |
| 8. | Line 144: Add exclusion criteria. | We really appreciate the valuable suggestion of worthy reviewer. Accordingly, the exclusion criterion has been added as suggested by the worthy reviewer. |
| 9. | Line 300: Need a reference. | With due respect, it is to highlight that perhaps worthy Reviewer is confusing the numerical data in line 300 with some historic data. Basically, the data presented in line 300 is findings of current study which has been mentioned here for discussion purposes and therefore we cannot add any reference here. |

**Journal requirements**

| **Sr. No.** | **Requirement** | **Response** |
| --- | --- | --- |
| **1.** | Please upload all main figures as separate Figure files in .tif or .eps format. For more information about how to convert and format your figure files please see our guidelines:  <https://journals.plos.org/plosntds/s/figures> | All figures have been formatted as required in afore-mentioned guideline. |
| **2.** | We have noticed that you have uploaded Supporting Information files, but you have not included a list of legends. Please add a full list of legends for your Supporting Information files after the references list. | Our supporting Information includes the raw data of study respondents in an anonymous manner. All the data presented in supporting information file is self-explanatory and no legends are required for this. |
| **3.** | Please ensure that the funders and grant numbers match between the Financial Disclosure field and the Funding Information tab in your submission form. Note that the funders must be provided in the same order in both places as well. | Verified and funding information has been added as per journal format. |
